# Quality of Life of Post-Mastectomy Women Living in a Semi-Arid Region of Brazil

**DOI:** 10.3390/ijerph14060601

**Published:** 2017-06-05

**Authors:** Emídio A. Araújo Neto, Beatriz C. A. Alves, Flávia de S. Gehrke, Ligia A. Azzalis, Virginia C. B. Junqueira, Luiz Vinicius de A. Sousa, Fernando Adami, Fernando L. A. Fonseca

**Affiliations:** 1 Laboratório de Análises Clínicas da Faculdade de Medicina do ABC, Av. Príncipe de Gales, 821, CEP 09060-650 Santo André, Brazil; clinicadremidioczpb@gmail.com (E.A.A.N.); bcaalves@uol.com.br (B.C.A.A.); flaviagehrke@hotmail.com (F.d.S.G.); 2 Departamento de Ciências Biológicas da Universidade Federal de São Paulo, UNIFESP, Rua Professos Artur Riedel, 275, CEP 09972-270 Diadema, Brazil; lazzalis@uol.com.br (L.A.A.); virginia.junqueira@gmail.com (V.C.B.J.); 3 Laboratório de Epidemiologia da Faculdade de Medicina do ABC, Av. Príncipe de Gales, 821, CEP 09060-650 Santo André, Brazil; luiz.sousa@fmabc.br (L.V.d.A.S.); adamifernando@uol.com.br (F.A.)

**Keywords:** mastectomy, breast cancer, neoplasia, quality of life, public health

## Abstract

Health is the major reference regarding quality of life; when it comes to breast cancer in particular, the loss of a breast traumatically affects a woman’s life, reflecting on her quality of life. Recognizing this problem, our aim was to investigate the quality of life of women who live in a semi-arid region of Brazil after breast cancer mastectomy. In this exploratory, transversal and observational study, a Brazilian variantof the shorter version of the original instrument from the World Health Organization Quality of Life (WHOQOL-BREF), applied in the study population, was analyzed and their socio-demographic profile was obtained. The sample was composed of 50 mastectomized women. The 50 included patients comprised women at a mean age of 54 years. Most of them had finished elementary school, and their average income was one Brazilian minimum monthly wage. Regarding the data related to quality of life, the highest score was found in the social relationships domain (4.29) followed by the psychological (4.09) and environmental (3.88) domains. The lowest score observed was for the physical domain (3.48). With these findings we can say that social and psychological parameters are driving factors of the quality of life in post-mastectomy women. Therefore, these results are useful to establish strategies to improve the quality of life of breast cancer mastectomy patients.

## 1. Introduction

Breast cancer is the most commonly diagnosed cancer among women both in developed and developing countries, and it is the major cause of death among those related to neoplasias. Around half a million women die worldwide as a consequence of the disease every year, making it a great concern for public health [1,2]. In Brazil, a total of 57,960 new cases are estimated for the year 2016, and in the state of Paraíba—located in a semi-arid region—the predicted number is 800 primary breast cancer cases [3].

There is a sharp increase in incidence rate up to the age of 50. This increase tends to slow down after this age, which may be related to the participation of female hormones in the etiology of the disease. However, breast cancer in young women has some clinical and epidemiological characteristics that differ remarkably from those in older women: in the former group, they are usually more aggressive with a high rate of gene mutations [3,4].

Concerning treatment, approaches like chemotherapy, hormone therapy, radiotherapy, and surgery should be taken into consideration [4]. Surgical procedures, once extremely aggressive, have recently become more and more ‘conservative’ given the improvement of techniques, introduction of new materials, better understanding of carcinogenesis, and knowledge on the evolution of neoplasias. Procedures like simple mastectomy (breast removal without lymph node resection), skin sparing mastectomy (preservation of as much as possible of breast skin without conservation of the nipple-areola complex), and nipple sparing mastectomy (preservation of skin and the nipple-areola complex) have been applied more and more these days.

Besides the total removal of the mammary gland parenchyma, procedures with only partial resection of the parenchyma are performed today. Quadrantectomy, for example, is a technique that involves the removal of a part of the breast that contains the tumor along with the skin that covers it. Segmentectomy is another method that consists of the excision of the tumor and part of the parenchyma with skin preservation. Tumorectomy (or lumpectomy) includes the resection of the tumor with a safety margin [4].

The improvement of some techniques and the recent development of new ones in clinical practice helped increase survival rates and decrease the after-effects in patients. Among them, one may mention the identification of the sentinel lymph node (gamma probe and vital dye staining techniques), the introduction of the nuclear magnetic resonance and morphofunctional analyses for the detection of metastasis (scintigraphy, Positron Emission Tomography/Computed Tomography-PET/CT, single photon emission computed tomography-SPECT), the localization of impalpable lesions (radio guided occult lesion localization, ROLL, and sentinel node and occult lesion localization, SNOLL), the introduction of immunohistochemical markers (estrogen receptor—ER, progesterone receptor—RP, and the oncogene HER2/neu, also known as C-ERB) in clinical practice routine, the discovery of new chemotherapeutics is increasingly effective with fewer side effects and the use of intraoperative radiotherapy (IORT) [5].

Health is the major reference regarding quality of life. For a long time, it was understood as the lack of diseases; however, as the concept of quality of life became broader, it was realized that, besides the absence of illnesses, there were other conditioning factors like education, leisure, living conditions, access to health services, nutritional standards, transportation and income, among others, that play an important role in people’s lives [6,7].

The World Health Organization, based on the The World Health Organization Quality of Life Group (WHOQOL), defined quality of life as “individuals‘ perception of their position in life in the context of the culture and value systems in which they live and in relation to their goals, expectations, standards, and concerns” [8].

Although many psychosocial effects of cancer are known, it is understood that the specter of the disease experience is vast, and it involves different moments with distinct meanings. When it comes to breast cancer in particular, the loss of a breast traumatically affects a woman’s life not only because it is a reproductive organ but also because it is one of the major symbols of femininity, thus reflecting on her quality of life.

Considering that mastectomy can be one of the factors that affect the quality of life of patients and that there are regional differences in Brazil, this study intends to show the differences in the health of a population determined by geographical and regional differences within the same context, Brazil, and determine whether or not these differences alter the quality of life after mastectomy.

Therefore, the objectives of this study were to analyze the quality of life of women after mastectomy due to breast cancer in Cajazeiras, a municipality in the state of Paraíba, Brazil, based on the results of the Brazilian variant of the shorter version of the original instrument from the World Health Organization Quality of Life WHOQOL-BREF [9] (an abbreviated version from WHOQOL-100 questionnaire) and to obtain their socio-demographic profile. Besides, we aimed to analyze the relation of quality of life in each domain with clinical characteristics.

## 2. Methods

An exploratory, transversal, and observational study was adopted. The sample was composed of 50 female participants with histopathological diagnosis of breast cancer. They all had undergone radical (Hasteld), modified (Patey and Madden), or partial (quadrantectomy or segmentectomy) mastectomy procedures, and they were registered in the Municipal Department of Health. All of the patients resided in Cajazeiras, Paraíba. Paraíba is one of the five poorest states in Brazil, with a disparate geographic area that includes the *Sertão* (semi-arid) and the coastal region. This state presents enormous difficulty of access to public health, the cases of cancer are underreported and there is great precariousness of the patients’ follow-up.

A Portuguese-translated version of the WHOQOL-BREF instrument was used. Upon answering the questions, in order to preserve spontaneity, privacy of the responses provided was assured to all patients.

Participants were indicated by the Nucleus of Family Assistance of the municipality of residence of each one of them. Inclusion criteria comprised women of any age group after at least three months of surgery so that there would be no post-operative clinical or psychological interference in the results. Exclusion criteria involved women who did not want to participate in the study, women with intellectual disabilities, and those who could not answer the questions due to lack of cognitive understanding. Interviews were conducted in the years of 2015 and 2016.

The project was approved by the Research Ethics Committee of the institution (CAAE# 48089115.0.0000.0082, under the number 077426/2015).

To describe the variations of the qualitative variables, the absolute frequency was used; for the quantitative variables with no normal distribution of the data Shapiro-Wilk (*p* < 0.05), median, 25th and 75th percentiles, minimum, and maximum were used; and for the variables with normal distribution Shapiro-Wilk (*p* > 0.05) was used to describe mean, standard deviation, minimum, and maximum.

For the analysis of the association of the psychological domain (a non-normal distribution was found with the Shapiro-Wilk test, *p* < 0.05) with mastectomy and the variable “breast reconstruction was performed”, the Kruskal-Wallis test was used; with the variable “type of therapy”, the Mann-Whitney test was applied. The confidence interval was of 95%.

Regarding the association of physical domain, social relationships, and environment (normal distribution, Shapiro-Wilk, *p* > 0.05) with the variables mastectomy and “breast reconstruction was performed”, the ANOVA test was used; with the variable “type of therapy”, the Student’s *t*-test was applied. The confidence interval was of 95%. The statistical package of choice was Data Analysis and Statistical Software for Professionals (Stata), version 11.0 (StataCorp LLC, College Station, TX, USA).

### Instruments

The following instruments were used for data collection: a socio-demographic, mammography-related questionnaire, and the WHOQOL-BREF questionnaire to evaluate the quality of life of the patients. The latter was developed by the World Health Organization (WHO) and translated and validated into the Portuguese language. Vital for the purpose, the questionnaire was administered by a trained interviewer. It includes 26 questions and uses a Likert-type scale, namely, it is subdivided into five options in which the patient is psychometrically evaluated. The score ranges from 1 to 5; for each question, the minimum score (1) is attributed if the patient totally disagrees with the statement, and the maximum score (5) is given if the patient totally agrees with it.

The first question of the WHOQOL-BREF is related to the individual perception concerning quality of life; the second, to the individual perception in relation to health. The other 24 questions are divided into four domains: physical, psychological, social relationships, and environment. The answers must be given in regard to situations that occurred two weeks prior to the day of the interview [10].

The physical domain is composed of the following facets disposition (tiredness or energy), discomfort and pain, day-to-day activities, productivity (ability to work), and rest. On the other hand, the psychological domain includes positivity, memory, concentration and learning ability, self-esteem, spirituality, and personal beliefs. The domain of social relations includes social relations and support as well as sexual activity. Finally, the environment domain measures physical safety and security, living and financial conditions, health care, quality of social spaces (public and private), opportunities to acquire new information and skills, participation in recreational and leisure activities, and the physical environment (pollution, noise, traffic, weather, and transportation) [9].

## 3. Results

The 50 female participants had a mean age of 54 years (range 51 to 61). As to the marital status, from a total of 50 women, 36% (*n* = 18) of the patients were married, 20% (*n* = 10) widowed, 16% (*n* = 8) unmarried, 12% (*n* = 6) separated, 10% (*n* = 5) divorced, and 6% had a different status.

Regarding education background, 4% of the participants were illiterate (people who cannot even write their own name), 8% could read and write, 24% had completed elementary school, 22% middle school, 18% high school, 18% had a college degree, and 6% (*n* = 3) had a higher educational level.

Most of the patients (52%, *n* = 26) reported having a per capita income of one Brazilian minimum monthly salary (930 Reais, present-day currency of Brazil, represents US$ 281), 20% (*n* = 10) from 1 to 3 salaries, 18% (*n* = 9) from 3 to 6 salaries, 6% (*n* = 3) from 6 to 10 salaries, and 4% (*n* = 2) had no income.

A total of 2% (*n* = 1) of the respondents lived alone, 2% (*n* = 1) with the father, 4% (*n* = 2) with the mother, 4% (*n* = 2) with a life partner, 32% (*n* = 16) with the spouse, 58% (*n* = 26) with their children, 12% (*n* = 6) with their grandchildren, and 32% (*n* = 16) in a different condition. When it came to the place where they lived, 66% (*n* = 33) were homeowners who had paid off their homes, 4% (*n* = 2) were homeowners still paying for their homes, 22% (*n* = 11) were renters, 6% (*n* = 3) lived in borrowed homes, and 2% (*n* = 1) lived in a different condition.

Concerning the number of live-birth pregnancies, 52% (*n* = 26) of the women had from one to three pregnancies, 28% (*n* = 14) more than three pregnancies and 20% (*n* = 10) never got pregnant.

In regard to breastfeeding, 40% (*n* = 20) never breastfed, 32% (*n* = 16) breastfed for less than six months and 28% (*n* = 14) for more than six months. In relation to the type of institution the mastectomy was performed, 54% (*n* = 27) of the cases were carried out in public institutions and 46% (*n* = 23) in private ones. The procedures are no different, but access to the public is more time-consuming than private, because of social inequality

When asked about how long ago the mastectomy had been performed, 18% (*n* = 9) of the respondents answered less than one year prior to the interview, 42% (*n* = 21) from 1 to 5 years, 12% (*n* = 6) from 5 to 10 years, and 28% (*n* = 14) over 10 years before the interview.

Regarding the data related to quality of life, when question number 1 (How would you rate your quality of life?) of the WHOQOL-BREF was analyzed, it could be observed that 74% of the respondents positively evaluated their quality of life (good or very good), 22% considered it intermediate (neither good nor bad), and only 4% negatively classified it (bad or very bad).

Question number 2 (How satisfied are you with your health?), related to health, was negatively answered by 4% of the patients (dissatisfied or very dissatisfied). On the other hand, 26% were undecided (neither satisfied nor dissatisfied), and 70% had a positive evaluation (satisfied or very satisfied).

The WHOQOL-BREF is based on a four-domain structure: physical, psychological, social relationships, and environmental. The mean score, on a scale from 1 to 5, was 3.48 for the physical domain, 4.09 for the psychological domain, 4.29 for the social and 3.88 for the environmental domain. Quality of life in general had a mean score of 3.34. Table 1 shows the characteristics of the quality of life of the samples.

Table 2 associates the clinical characteristics with the physical domain. As observed, patients who underwent unilateral or conservative mastectomy had the same score. As to breast reconstruction, those who had the breast implant inserted during the mastectomy procedure had a higher score. Regarding the treatment performed, patients who went through either chemotherapy or radiotherapy and those who underwent only hormone therapy scored higher than the others.

According to the association shown in Table 3 (social relationships and clinical characteristics), patients who underwent conservative mastectomy had a higher score, as well as those who had the breast reconstruction procedure. Regarding the type of treatment, patients who underwent both chemotherapy and radiotherapy scored higher.

The relation between the environmental domain and the clinical characteristics (Table 4) showed a higher score among patients who underwent unilateral mastectomy and immediate breast reconstruction. Those who were treated with chemotherapy only had a higher score in relation to this domain with this type of treatment. Table 4 shows the association between the environmental domain and the clinical characteristics.

## 4. Discussion

Our results showed that Cajazeiras women who underwent mastectomy had median quality of life scores in the psychological and social relationships domains close to the maximum (between 4 and 5). On the other hand, in the physical and environmental domains they reached an intermediate median score (between 3 and 4). According to Sousa et al. (2014) [11], all four domains had an intermediate median score, and the psychological domain got the highest median score (14.76 on a scale from 4 to 20). Physical, social relationships, and environment got 12.15, 12.80, and 12.33 respectively. In the current study, the most compromised score was in the physical domain (3.48), which corroborates the result found in Brasília.

In Cajazeiras, the facets with the lowest median scores were pain and discomfort, reported by most of the participants as hindering factors. Regarding sleep and rest, the respondents declared being neither satisfied nor dissatisfied. They stated being dissatisfied when it came to the ability to perform daily routine activities and work.

According to Sampaio (2006) [12], motor difficulties and other aspects are limitations imposed by the condition. Issues like the reduction in the range of movement of the arm on the side where the surgery was performed, pain, and discomfort are restraining factors in regular daily activities. Besides, as pain is a distressing and negative sensation, it brings out stress and suffering, thus affecting the quality of life.

Canário et al. (2016) [13] conducted a study in the state of Rio Grande do Norte, which corroborates our findings. A high prevalence of the symptoms mentioned above was found, therefore affecting the physical domain.

Social relationships had the best median score (4.29); it is composed of social relations, social support, and sexual life. Hence, it is possible to imply that the participants have good personal relations, and they feel socially supported by family members, friends, relatives, or acquaintances. Interestingly, even those who did not have a life partner obtained a high median score in this domain, thus showing that the presence or absence of a life partner does not alter this parameter.

Breast cancer and mastectomy are associated with many beliefs, symbologies, and stigmas that haunt women’s thoughts in their daily lives. Depending on the kind of relationship a post-mastectomy woman will have with her life partner, her quality of life may go through deep changes. Life partners of women with breast cancer may be a source of emotional support or stress, resulting in a positive or negative influence on these patients’ quality of life [14,15,16]. According to Greendale et al. (2001) [17], women who are pleased with their partners declared being psychologically well-adjusted and sexually satisfied.

In the current study, when social relationships domain was compared with the type of surgery patients went through, those who had undergone conservative mastectomy had the highest median score. This result confirms not only the findings of Kluthcovsky et al. (2012) [18] in the state of Paraná, in which the highest median score for quality of life was in the social relationships domain, but also the results found by Al-Ghazal et al. (2000) [19], Amivhetti & Caffo (2001) [20], and Yan et al. (2016) [21].

It is important to point out that, although the psychological domain obtained a high median score (4.09), the facet with the lowest values was the physical appearance. Sousa et al. (2014) [11] found the same results in a study conducted in Brasília, in which 60% of the patients had difficulty accepting their appearance, especially those who did not undergo breast reconstruction. Beauty, a concept molded by society, is the parameter that most influences a woman’s body image; therefore, the preservation of the ‘I-concept’ in patients becomes very difficult.

The environmental domain scored 3.88. It could be observed that many women are very satisfied with the place where they live, but they expressed dissatisfaction (score 1) regarding leisure opportunities. In Sousa et al.’s work (2014) [11], the environmental domain also had an average score, and the lowest median scored facet was that related to safety.

The facet “satisfaction with the place where you live” scored high in our results (4 and 5), which leads to the conclusion that, despite the fact our patients were from the Brazilian *Sertão*, the semi-arid region in Northeastern Brazil, the perception in relation to the quality of life did not change. In fact, it was confirmed with the data associated with the environmental domain, i.e., the *Sertão* does not improve or worsen the studied condition.

Both Huguet et al. (2009) [15], in the study on the quality of life of under-treatment breast cancer patients in Campinas, São Paulo, and Amaral et al. (2009) [16], when researching post-mastectomy women in outpatient chemotherapy treatment in the state of Alagoas, revealed that women from a higher socioeconomic status scored higher on the environmental domain.

Our results show that income did not influence on the facet “satisfaction with the place where you live”. One of the possible conclusions is that the concept of being wealthy may be different, financially speaking, when a big city lifestyle is compared with the lifestyle in small town in the *Sertão*. Our sample was composed of 50 patients, a relatively small number, but a very representative set in proportion to the town’s population. The focus of the current study was on the women from the Brazilian *Sertão*, more specifically, on those from the Cajazeiras municipality. However, due to the lack of statistical significance, results here described should be taken with reservation.

The theme may be of great relevance to health care management and all health professionals involved in the treatment of breast cancer and post-mastectomy patients since very little is discussed about the latter topic.

## 5. Conclusions

It is of utmost importance to evaluate the quality of life of post-mastectomy patients and analyze how much suffering is involved after the procedure. Furthermore, their mental and physical well-being, their family and social relationships, as well as education and other factors that might interfere with their integrity should be taken into account. The findings of this study show many factors that directly interfere with the quality of life of post-mastectomy women who live in the Cajazeiras municipality. The physical factor was highlighted, as pain and the reduction in the range of movement of the arm on the side where the surgery was performed were frequently reported by the participants. Regarding the social aspect, all the support provided by family members, and those who are close to these women, are driving factors for a better quality of life. The psychological factor should be dealt with by skilled professionals due to the fact the breast is an organ full of symbology for women.

Finally, when it came to the environmental factor, the research team reached the conclusion that living in a small town in the Brazilian *Sertão* does not interfere with the quality of life of mastectomized women.

## Figures and Tables

**Table 1:** Characteristics of quality of life of the sample.

Domains of WHOQOL-BREF	Mean (sd)	Minimum–Maximum
Physical domain	3.5 (0.9)	1.2–4.8
Social relationships	4.2 (0.4)	3.3–5.0
Environment	3.8 (0.6)	2.8–4.8
	Median (p. 25–p. 75)	
Psychological domain	4.2 (3.8–4.7)	2.2–5.0

Note: sd: Standard deviation; p. 25–p. 75: Percentile 25 to percentile 75. WHOQOL-BREF is a shorter version of the original instrument from the World Health Organization Quality of Life.

**Table 2:** Association between the physical domain and the clinical characteristics. (Line 191, Results).

Variables	*n* (%)	Physical Domain	
		Mean (95% CI)	p *
Total or conservative mastectomy			
Total unilateral	40 (80.0)	3.5 (3.4; 3.9)	0.117
Total bilateral	6 (12.0)	2.8 (2.0; 3.7)	
Conservative	4 (8.0)	3.5 (2.6; 4.4)	
Breast reconstruction			
Yes, immediate reconstruction	10 (20.0)	3.6 (3.1; 4.3)	0.617
Yes, delayed reconstruction	1 (2.0)	2.8 (2.8; 2.8)	
No	39 (78.0)		
Type of adjuvant treatment			p **
Chemotherapy			
No	40 (80.0)	3.6 (3.2; 3.8)	0.568
Yes	10 (20.0)	3.4 (2.7; 3.9)	
Chemotherapy and radiotherapy			
No	20 (40.0)	3.3 (2.9; 3.7)	0.116
Yes	30 (60.0)	3.7 (3.3; 4.0)	
Radiotherapy			
No	46 (92.0)	3.4 (3.2; 3.8)	0.899
Yes	4 (8.0)	3.5 (2.1; 5.0)	
Hormone therapy			
No	15 (30.0)	3.2 (2.7; 3.6)	0.092
Yes	35 (70.0)	3.7 (3.4; 3.9)	
None			
No	46 (92.0)	3.6 (3.2; 3.8)	0.179
Yes	4 (8.0)	2.9 (1.0; 4.9)	

* ANOVA; ** Student’s *t*-test. 95% CI: 95% Confidence Interval.

**Table 3:** Association between social relationships domain and clinical characteristics. (Line 196, Results).

Variables	*n* (%)	Social Relationships	
		Mean (95% CI)	*p* *
Total or conservative mastectomy			
Total unilateral	40 (80.0)	4.3 (4.1; 4.4)	0.332
Total bilateral	6 (12.0)	4.1 (3.4; 4.8)	
Conservative	4 (8.0)	4.5 (3.7; 5.4)	
Breast reconstruction			
Yes, immediate reconstruction	10 (20.0)	4.2 (4.0; 4.5)	0.384
Yes, delayed reconstruction	1 (2.0)	3.7	
No	39 (78.0)	4.4 (4.1; 4.5)	
Type of adjuvant treatment			p **
Chemotherapy			
No	40 (80.0)	4.4 (4.2; 4.5)	0.164
Yes	10 (20.0)	4.1 (3.7; 4.5)	
Chemotherapy and radiotherapy			
No	20 (40.0)	4.1 (3.9; 4.2)	0.003
Yes	30 (60.0)	4.5 (4.3; 4.7)	
Radiotherapy			
No	46 (92.0)	4.3 (4.2; 4.4)	0.376
Yes	4 (8.0)	4.0 (3.9; 4.4)	
Hormone therapy			
No	15 (30.0)	4.1 (3.9; 4.3)	0.030
Yes	35 (70.0)	4.4 (4.3; 4.6)	
None			
No	46 (92.0)	4.3 (4.1; 4.4)	0.214
Yes	4 (8.0)	4.0 (3.2; 4.8)	

* ANOVA; ** Student’s *t*-test. 95% CI: 95% Confidence Interval.

**Table 4:** Association between the environmental domain and the clinical characteristics.

Variables	*n* (%)	Environment	
		Mean (95% CI)	p *
Total or conservative mastectomy			
Total unilateral	40 (80.0)	4.0 (3.8; 4.1)	0.210
Total bilateral	6 (12.0)	3.7 (3.0; 4.3)	
Conservative	4 (8.0)	3.7 (3.0; 4.4)	
Breast reconstruction			
Yes, immediate reconstruction	10 (20.0)	4.2 (3.9; 4.4)	0.074
Yes, delayed reconstruction	1 (2.0)	3.0	
No	39 (78.0)	3.9 (3.6; 4.1)	
Type of adjuvant treatment			p **
Chemotherapy			
No	40 (80.0)	3.9 (3.7; 4.0)	0.271
Yes	10 (20.0)	4.1 (3.7; 4.4)	
Chemotherapy and radiotherapy			
No	20 (40.0)	3.9 (3.7; 4.1)	0.980
Yes	30 (60.0)	3.8 (3.6; 4.1)	
Radiotherapy			
No	46 (92.0)	3.9 (3.7; 4.1)	0.472
Yes	4 (8.0)	3.7 (2.8; 4.5)	
Hormone therapy			
No	15 (30.0)	3.9 (3.5; 4.2)	0.778
Yes	35 (70.0)	3.9 (3.7; 4.1)	
None			
No	46 (92.0)	3.9 (3.8; 4.0)	0.971
Yes	4 (8.0)	3.9 (3.3; 4.5)	

* ANOVA; ** Student’s *t*-test. 95% CI: 95% Confidence Interval.
